# Aortic Pseudoaneurysm Secondary to Mediastinitis due to Esophageal Perforation

**DOI:** 10.1155/2016/7982641

**Published:** 2016-02-10

**Authors:** Claudia Patricia Zuluaga, Felipe Aluja Jaramillo, Sergio Andrés Velásquez Castaño, Aura Lucía Rivera Bernal, Julio Cesar Granada, Jorge Alberto Carrillo Bayona

**Affiliations:** ^1^Hospital Universitario Mayor, Mederi, Bogotá, Colombia; ^2^Fundación Universitaria Sanitas, Bogotá, Colombia; ^3^Universidad del Rosario, Bogotá, Colombia

## Abstract

Esophageal perforation is a condition associated with high morbidity and mortality rates; it requires early diagnosis and treatment. The most common complication of esophageal rupture is mediastinitis. There are several case reports in the literature of mediastinitis secondary to esophageal perforation and development of aortic pseudoaneurysm as a complication. We report the case of a patient with an 8-day history of esophageal perforation due to foreign body (fishbone) with mediastinitis and aortic pseudoaneurysm. The diagnosis was made using Computed Tomography (CT) with intravenous and oral water-soluble contrast material. An esophagogastroduodenoscopy did not detect the perforation.

## 1. Case Report

A 54-year-old female patient was admitted to the emergency department with an 8-day history of epigastric pain that began one day after eating fish. She consulted at another institution five days before, where she underwent esophagogastroduodenoscopy that did not reveal any foreign body or esophageal abnormalities. The symptoms got worse despite antacids and analgesic therapy so she consulted at our institution. She has a personal history of type 2 diabetes mellitus. The physical examination revealed tachycardia and intense epigastric pain on palpation.

The hepatic biochemistry and blood amylase levels were within the normal range. A complete blood count documented leukocytosis (18.500 cells/mm^3^) with neutrophilia (85.3%) and positive C-reactive protein (161.8 mg/L).

**Figure 1:**
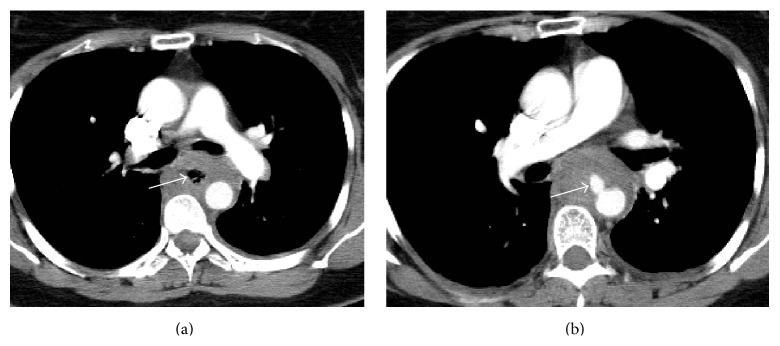
(a) Axial enhanced CT scan. Mediastinal collection with air bubbles within it (white arrow). (b) Axial contrast enhanced CT scan. Pseudoaneurysm of the thoracic aorta (white arrow).

Contrast enhanced thoracic and abdominal Computed Tomography was performed. The CT scan showed the presence of a pseudoaneurysm of the thoracic aorta, thickening of the esophageal wall, and abnormal density of the mediastinal fat with air bubbles within it that suggested mediastinitis. There was no evidence of contrast material extravasation from the esophageal lumen (Figure 1).

**Figure 2:**
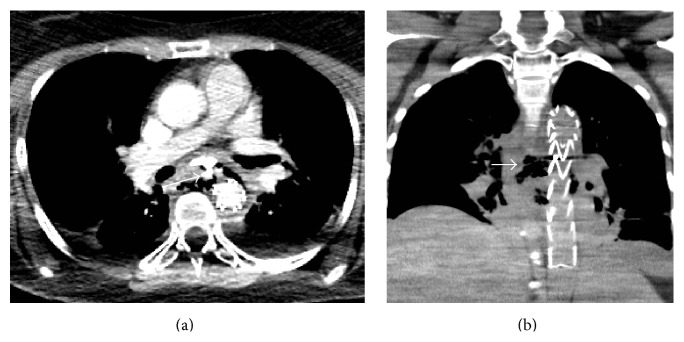
CT scan using oral hydrosoluble contrast material: (a) axial and (b) coronal reconstruction. (a) There is leakage of the contrast material from the esophagus to the posterior mediastinum (white arrow); the aortic stent was placed demonstrating that the esophageal wall is perforated. (b) Mediastinal collection with air bubbles within it (white arrow), surrounding the aorta with the stent placed in an adequate position.

An aortic endoprosthesis was placed and a second CT scan was performed using oral hydrosoluble contrast material. Leakage of the contrast material to the posterior mediastinum, approximately 6 cm below the carina, was clearly seen (Figure 2).

A second esophagogastroduodenoscopy confirmed an esophageal perforation. An esophageal stent was placed. The patient was then taken to surgery (right posterolateral thoracotomy) to drain the mediastinitis, debride the necrotic tissue, and perform transposition of a pedicled intercostal muscle flap to cover the esophageal defect. The patient had a satisfactory evolution.

## 2. Discussion

Esophageal perforations can be spontaneous or secondary to trauma, iatrogenic lesions, foreign body ingestion, and tumoral processes [1, 2]. The presence of foreign bodies is a frequent condition [3]. Ingested sharp-pointed objects lodged in the esophagus are a medical emergency. These elements may pass through the esophagus without affecting the esophageal structure (80% of the cases) but 10 to 20% of ingested foreign bodies will require endoscopic removal [4, 5]. Ingested foreign bodies are responsible for 80% of cervical perforations [6]. Fish bones are a predominant cause (60%) [7–9], followed by chicken bones (16%) and other objects such as coins [9, 10]. Perforation occurs in up to 4% of those patients [3, 7, 8, 11], with 22% mortality according to a series of 511 patients [1, 11, 12] and 20% according to Brinster and colleagues [13].

Another less frequent cause of esophageal perforation (0.25%) is those lesions that occur during endoscopic removal of ingested foreign bodies.

Esophageal perforations due to ingestion of a foreign body can cause complications like mediastinal infection, vascular trauma (aortoesophageal fistula, pseudoaneurysm), paraesophageal abscess, tracheoesophageal fistula, pneumomediastinum, pneumothorax, pericarditis, and some others [10, 11, 14–17]. Foreign bodies may migrate to adjacent structures including the thyroid gland [9] forming abscesses in the deep neck [7].

Vascular structures such as the aorta, subclavian artery, internal carotid artery, and the internal jugular vein [7] may be affected [1]. The lesions that affect the aortic wall may be secondary to direct puncture of the wall by the foreign body or due to the extension of the mediastinal inflammatory process with an aortic rupture contained by the adjacent soft tissues and the inflammatory exudate (pseudoaneurysm). In some cases there can be direct communication between the esophagus and the aorta (aortoesophageal fistula) [3, 7]. Eventually, the site of esophageal perforation is occluded by a clot and hematoma that leads to partial tamponade that prevents subsequent bleeding [3].

The most common site of aortic complications is located 1 to 5 cm distal to the origin of the left subclavian artery [18–20]. These vascular complications have severe mortality and morbidity [7, 18, 19], which may develop after weeks or years with an invariable fatal ending [11].

In order to compile this review article we conducted a selective literature research in PubMed. 81 articles in English matched our search terms “foreign body ingestion AND mediastinitis, aortic pseudoaneurysm AND aortoesophageal fistula”. After excluding articles of aortoesophageal fistula, we were left with 8 case reports. All of these cases had clinical features and imaging findings of mediastinitis with secondary aortic pseudoaneurysm. The causes of perforation were fish bone ingestion as the most common, followed by chicken bone ingestion and one case of complication after esophageal botulinum toxin injection for achalasia. All patients consulted with similar symptoms including hematemesis, fever, dysphagia, odynophagia, and systemic inflammatory response syndrome. All consulted the emergency room 3 to 12 days after the perforation occurred (Table 1).

Radiographic studies are necessary to confirm the diagnosis of foreign bodies and esophageal perforation. Perforation of the cervical esophagus can lead to the presence of prevertebral air bubbles and soft tissue thickening. In 90% of thoracic esophagus perforation presence of pleural effusion, pneumothorax and hydropneumothorax can be seen [6, 21]. Right-sided pleural effusion occurs if the perforation is located in the middle third of the esophagus. Left-sided pleural effusions are commonly associated with distal esophageal perforation. Esophageal perforation may also be diagnosed by the presence of food particles, pH less than 6.0, or the presence of an elevated amylase level in the pleural fluid analysis [6, 21].

In the setting of foreign bodies, CT scan is useful in those cases with suspected complications (e.g., perforation) or when it is necessary to identify the foreign body before esophagogastroduodenoscopy [5]. Cervical, thoracic, and abdominal CT scan with oral and intravenous contrast is currently the modality of choice in the assessment of esophageal perforation with a sensitivity of 92 to 100% [6]. This imaging method allows evaluation of the extent of the perforation based on associated findings such as mediastinitis, pleural effusion, extrapleural collections, and intraperitoneal effusion [6]. Extraluminal air is the most common CT finding in esophageal perforation, occurring in almost 92% of cases [21].

Esophagography is useful in identifying the perforation when extravasation of hydrosoluble contrast material occurs allowing also establishing if the leak is contained or not [6]. It has a sensitivity of 50% for the detection of cervical esophageal perforation and of 75 to 80% for the detection of thoracic esophageal perforation; however, it has an overall false-negative rate of 10% [6]. If the first esophagography is negative but there are strong clinical signs and symptoms of perforation or a suspicion of a fistulous tract, a second esophagography is advised in the following hours [6].

Esophagogastroduodenoscopy has a sensitivity of nearly 100% and a specificity of 83% for the detection of esophageal perforation [21]. However, it is not recommended as the primary diagnostic method in esophageal perforation because it may miss a perforation hidden in a mucosal fold and has the potential to enlarge a small mucosal or submucosal tear turning it into a large perforation during air insufflation process [21].

As relevant facts in our case, the initial esophagogastroduodenoscopy and esophageal CT scan did not show an esophageal perforation. Although the initial vascular complication was managed, the alterations associated with the mediastinitis made a second CT evaluation mandatory, which showed the perforation and enabled the appropriate treatment of the underlying condition (esophageal perforation with secondary mediastinitis).

## 3. Conclusion

Among the possible complications of mediastinitis secondary to esophageal perforation, vascular lesions must be considered (pseudoaneurysm and aortoesophageal fistula), which have a high morbimortality. Oral and intravenous enhanced CT is the study of choice to diagnose esophageal perforation and identify associated complications.

## Figures and Tables

**Table 1:** Foreign body and false aneurysm PubMed search results.

Authors	Country	Sex	Age	Cause of perforation	*T*	Signs and symptoms	Imaging findings	Treatment	Follow-up
Chao et al. [2]	Australia	M	76 y	Esophageal botulinum toxin injection for achalasia	1 week	Chest pain, SIRS	Pseudoaneurysm of the descending aorta with mediastinal abscess	Endovascular stent graft, antibiotics	Discharge, follow-up for 4 months with no symptoms

Chen et al. [14]	China	F	57 y	Fish bone	6 days	Chest discomfort, chills, emesis, dysphagia, SIRS	Pseudoaneurysm in the aortic isthmus, mediastinal abscess, bilateral pleural effusion	Antibiotics, resection of the pseudoaneurysm, resection of the esophagus	Discharge, but suicide a year later

Chen et al. [14]	China	M	54 y	Fish bone	6 days	Chest pain, hematemesis, SIRS	Pseudoaneurysm in the aortic isthmus, mediastinal abscess, bilateral pleural effusion	Endovascular stent graft, antibiotics, bilateral thoracostomy	Discharge, follow-up for 2 months with no symptoms

Choi et al. [22]	South Korea	M	31 y	Fish bone	3 days after fish bone removal	Fever	Aortic rupture	Endovascular stent graft, esophageal resection	Discharge, follow-up for 4 months with no symptoms

Chen et al. [23]	China	M	22 y	Chicken bone	1 week	Chest discomfort, hematemesis, leukocytosis	Esophageal-mediastinal fistula surrounded by inflammatory exudate Pseudoaneurysm of the descending aorta	Endovascular stent graft, esophageal stent, mediastinal debridement	Discharge, the esophageal stent was removed 80 days after surgery; follow-up for 6 months with no symptoms

Kunishige et al. [1]	Japan	F	79 y	Fish bone	11 days after fish bone removal	Hematemesis, SIRS, positive PCR	Pseudoaneurysm of the aortic arch with no fistulous tract with the esophagus	Esophageal hemostasia, antibiotics, thoracotomy with mediastinal debridement; the space was filled with omentum from colon and stomach	Discharge, follow-up for 2 months with no symptoms

Sia et al. [24]	Malaysia	M	54 y	Fish bone	1 week	Hematemesis, odynophagia, dysphagia, fever	Saccular outpouching (pseudoaneurysm) of the descending aorta Mediastinal abscess	Endovascular stent graft, antibiotics	Esophageal reconstruction was not possible because the patient died due to sepsis

Sica et al. [17]	United Kingdom	F	57 y	Fish bone	1 week	Chest pain, SIRS, positive PCR	Abscess in the superior mediastinum, contrast leakage from the aorta into the mediastinum	Antibiotics, thoracotomy with debridement of the mediastinal tissue, aortic homograft patch, resection of the esophagus	Discharge, follow-up for 12 months with no symptoms

*T*: time from perforation to emergency room.

## References

[1] Kunishige H., Myojin K., Ishibashi Y., Ishii K., Kawasaki M., Oka J. (2008). Perforation of the esophagus by a fish bone leading to an infected pseudoaneurysm of the thoracic aorta. *General Thoracic and Cardiovascular Surgery*.

[2] Chao C. Y., Raj A., Saad N., Hourigan L., Holtmann G. (2015). Esophageal perforation, inflammatory mediastinitis and pseudoaneurysm of the thoracic aorta as potential complications of botulinum toxin injection for achalasia. *Digestive Endoscopy*.

[3] Bullaboy C. A., Derkac W. M., Johnson D. H., Jennings R. B. (1985). False aneurysm of the aorta secondary to an esophageal foreign body. *The Annals of Thoracic Surgery*.

[4] Ambe P., Weber S. A., Schauer M., Knoefel W. T. (2012). Swallowed foreign bodies in adults. *Deutsches Arzteblatt International*.

[5] Young C. A., Menias C. O., Bhalla S., Prasad S. R. (2008). CT features of esophageal emergencies. *RadioGraphics*.

[6] Chirica M., Champault A., Dray X. (2010). Esophageal perforations. *Journal of Visceral Surgery*.

[7] Ko S.-F., Lu H.-I., Ng S.-H., Kung C.-T. (2013). Fishbone penetration of the thoracic esophagus with prolonged asymptomatic impaction within the aorta. *Journal of Vascular Surgery*.

[8] Ngan J. H. K., Fok P. J., Lai E. C. S., Branicki F. J., Wong J. (1990). A prospective study on fish bone ingestion: experience of 358 patients. *Annals of Surgery*.

[9] D'Costa H., Bailey F., McGavigan B., George G., Todd B. (2003). Perforation of the oesophagus and aorta after eating fish: an unusual cause of chest pain. *Emergency Medicine Journal*.

[10] Dahiya M., Denton J. S. (1999). Esophagoaortic perforation by foreign body (coin) causing sudden death in a 3-year-old child. *American Journal of Forensic Medicine & Pathology*.

[11] Lam E. C. S., Brown J. A., Whitaker J. S. (2003). Esophageal foreign body causing direct aortic injury. *Canadian Journal of Gastroenterology*.

[12] Jones W. G., Ginsberg R. J. (1992). Esophageal perforation: a continuing challenge. *The Annals of Thoracic Surgery*.

[13] Brinster C. J., Singhal S., Lee L., Marshall M. B., Kaiser L. R., Kucharczuk J. C. (2004). Evolving options in the management of esophageal perforation. *Annals of Thoracic Surgery*.

[14] Chen A.-P., Yu H., Li H.-M., Xiao X.-S., Liu S.-Y. (2011). Aortoesophageal fistula and aortic pseudoaneurysm induced by swallowed fish bone: a report of two cases. *CardioVascular and Interventional Radiology*.

[15] Giménez A., Franquet T., Erasmus J. J., Martínez S., Estrada P. (2002). Thoracic complications of esophageal disorders. *RadioGraphics*.

[16] Macchi V., Porzionato A., Bardini R., Parenti A., De Caro R. (2008). Rupture of ascending aorta secondary to esophageal perforation by fish bone. *Journal of Forensic Sciences*.

[17] Sica G. S., Djapardy V., Westaby S., Maynard N. D. (2004). Diagnosis and management of aortoesophageal fistula caused by a foreign body. *Annals of Thoracic Surgery*.

[18] Bathla G., Teo L. L. S., Dhanda S. (2011). Pictorial essay: complications of a swallowed fish bone. *Indian Journal of Radiology and Imaging*.

[19] Leow C. K. (1998). Subclavian arterio-esophageal fistula secondary to fish bone impaction: report of a case. *Surgery Today*.

[20] Sloop R. D., Thompson J. C. (1967). Aorto-esophageal fistula: report of a case and review of the literature. *Gastroenterology*.

[21] Wu J. T., Mattox K. L., Wall M. J. (2007). Esophageal perforations: new perspectives and treatment paradigms. *The Journal of Trauma—Injury, Infection and Critical Care*.

[22] Choi J., Lee S., Moon J., Choi H. (2009). Fish bone induced aortic rupture treated with endovascular stent graft. *European Journal Cardio-Thoracic Surgery*.

[23] Chen X., Li J., Chen J. (2012). A combined minimally invasive approach for the treatment of aortoesophageal fistula caused by the ingestion of a chicken bone: case report and literature review. *Clinics*.

[24] Sia K. J., Ashok G. D., Ahmad F. M. A., Kong C. K. L. (2013). Aorto-oesophageal fistula and aortic pseudoaneurysm caused by a swallowed fish bone. *Hong Kong Medical Journal*.

